# Calculation of Blood Dose in Patients Treated With ^131^I Using MIRD, Imaging, and Blood Sampling Methods

**DOI:** 10.1097/MD.0000000000003154

**Published:** 2016-03-18

**Authors:** Elham Piruzan, Mahdi Haghighatafshar, Reza Faghihi, Seyed Mohammad Entezarmahdi

**Affiliations:** From the Nuclear Medicine and Molecular Imaging Research Center (EP, MH, SME), Namazi Teaching Hospital, Shiraz University of Medical Sciences; Ray Medical Engineering Department (EP, RF), Shiraz University, Shiraz, Iran; Radiation Research Center (RF), Shiraz University, Shiraz, Iran; and Radiation Medicine Engineering Department (SME), Shahid Beheshti University, GC, Tehran, Iran.

## Abstract

Radioiodine therapy is known as the most effective treatment of differentiated thyroid carcinoma (DTC) to ablate remnant thyroid tissue after surgery. In patients with DTC treated with radioiodine, internal radiation dosimetry of radioiodine is useful for radiation risk assessment. The aim of this study is to describe a method to estimate the absorbed dose to the blood using medical internal radiation dosimetry methods.

In this study, 23 patients with DTC with different administrated activities, 3.7, 4.62, and 5.55 GBq after thyroidectomy, were randomly selected. Blood dosimetry of treated patients was performed with external whole body counting using a dual-head gamma camera imaging device and also with blood sample activity measurements using a dose calibrator. Absorbed dose to the blood was measured at 2, 6, 12, 24, 48, and 96 hours after the administration of radioiodine with the 2 methods.

Based on the results of whole body counting and blood sample activity dose rate measurements, 96 hours after administration of 3.7, 4.62, and 5.55 GBq of radioiodine, absorbed doses to patients’ blood were 0.65 ± 0.20, 0.67 ± 0.18, 0.79 ± 0.51 Gy, respectively. Increasing radioiodine activity from 3.7 to 5.55 GBq increased blood dose significantly, while there was no significant difference in blood dose between radioiodine dosages of 3.7 and 4.62 GBq.

Our results revealed a significant correlation between the blood absorbed dose and blood sample activity and between the blood absorbed dose and whole body counts 24 to 48 hours after the administration of radioiodine.

## INTRODUCTION

Many people suffer from thyroid cancer annually and differentiated thyroid carcinoma (DTC) is the most common type.^1^ Radioiodine therapy is known as an effective treatment of DTC to ablate remnant thyroid tissue after surgery and to treat iodine-avid metastases.^2^ The different aspects of radioiodine therapy including methods, benefits, and risks can be found in the European Association of Nuclear Medicine (EANM) guidelines and textbooks.^2^ Organ absorbed doses and estimation of radiation risk is a major challenge in nuclear medicine. As external radiation dosimetry,^3^ internal radiation dosimetry of radiopharmaceuticals is an important aspect of radiation risk assessment and calculation of maximum tolerable activity.

Radiation absorbed dose to the blood, red bone marrow, and most organs, in the treatment of DTC with radioiodine, cannot be measured directly.^4^ It has been demonstrated that the dose in bone marrow and blood are the same in this procedure.^5^ The measurement of absorbed dose to the blood seems to be an appropriate estimation of the radiation absorbed dose to the hematopoietic system and provides a better understanding of the treatment quality.^6^ One of the pioneer blood dosimetry methods was introduced by Benua et al,^7^ and they presented a method to calculate the tolerated dose to blood and also the dose to the target per unit of administered activity. They showed that radioiodine therapy is safe provided that the blood dose is less than 2 Gy (200 rad), the whole body retention less than 4.4 GBq (120 mCi) at 48 hours, and also the pulmonary uptake at 24 hours less than 3 GBq (80 mCi). The absorbed dose to the blood per unit activity administered, depends on the patient's weight and renal clearance, and can be changed by a factor of more than 5.^8^ In 2006, Tuttle et al^9^ showed that for elderly patients treated during hypothyroidism, after administration of activities equivalent or less than 7.4 GBq (200 mCi), the blood dose exceeds 2 Gy. On the other hand, very low administered activity might lead to reduced radioiodine for target tissue uptake. Whereas in blood absorbed dose component model calculation, there is an almost linear relation between the blood and the whole body residence time which is the major determinant of dose to the blood, whole body, and target tissue.^9^ In the relevant guidelines, blood dosimetry is recommended as part of standard operational procedures, except if the dose to the blood does not exceed 2 Gy.^2^ In another study, the blood dose was estimated from a single external measurement of the whole body retention 1 or 2 days after radioiodine administration.^10^ Determining the standard amount of radioiodine activity is an important aspect for an optimally successful treatment. In a recent investigation, the desirable activity values ranging from 0.99 to 3.7 GBq with acceptable results in the ablation of tissue in DTC cases are reported.^11^ Higher activity is recommended for metastatic cases but, these can lead to serious risk to bone marrow and healthy tissues, therefore activity values are limited to around 7.4 GBq.^12,13^ The aim of this study is to describe a method to estimate the dose to the blood and maximum tolerable activity using medical internal radiation dosimetry (MIRD) methods, and to find the correlation between absorbed dose to the blood, blood sample activity, and whole body counts at an appropriate time after administrated activity.

## MATERIAL AND METHODS

Twenty three patients, 20 women and 3 men, suffering from DTC were enrolled in this study. The patients did not have renal failure, gastrointestinal disease, and did not use diuretics. As the thyroid cancer is more common among women, 86.9% of the patients included in this study are female. The necessary specifications were obtained from patients’ documents. Patients were selected randomly with different administration activities; 3.7, 4.62, and 5.55 GBq after thyroidectomy. According to MIRD instructions, in this study the blood dosimetry was performed using whole body imaging and blood sampling methods. For dosimetry in the blood-based method, radioactivity of 2 important components needs to be monitored: blood and whole body. The activity in the whole body was measured by conjugate views of whole body imaging with a dual-head gamma camera using a fixed geometry, and blood sample activity was measured with dose calibrator. According to the guidelines of the Dosimetry Committee of EANM, the details of the blood sampling frequency of the data and dose calculation procedure and quality control were performed. Our study was approved by the Institutional Review Board, and informed consent was waived.

### Whole Body Scintigraphy Conjugate-View Imaging

Whole body measurements were performed as conjugate view (anterior and posterior) counts by calibrated General Electric Infinia Hawkeye 4 scintillation dual-head gamma camera imaging after each administration of radioiodine. Planar gamma camera images were analyzed by extraction of count rates registered in regions of interest including the entire body. All imaging was performed with fixed patient positions and the same distance between patient and heads of gamma camera. The collimators of gamma camera, acquisition, and geometrical settings were the same for all the scans. Also, all imaging was done with fixed speed 130 mm/min and the same imaging duration. Imaging was performed at 6 periods, 2, 6, 12, 24, 48, and 96 hours after administrated activity for each patient. Background counts for 2 minutes were measured before each imaging, and background correction was performed for all whole body scans. So, geometric mean of anterior and posterior counts was calculated for each patient separately. Retention was estimated by normalization of the geometric mean of background corrected anterior and posterior counts to the initial measurement which was made 2 hours after administration without interim maturation or defecation. According to standard operational procedure in the case of oral administration, the urinary bladders of the patients should be emptied before the activity is administrated, the patients were asked to endure without maturation, the 1st 2 hours after administration, while maturation is compulsive before all subsequent whole body measurements (6, 12, 24, 48, and 96 hours). To determine the mean background counts per pixel (BKG_cts/pix_), the background scan was done. The whole body counts (WB_cts_) and number of pixels (WB_pix_) in the whole body ROI were extracted, and the whole body net counts (WB_net_) were obtained according to the following formula (2): 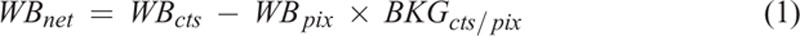


Also, geometric mean was calculated by using the following formula (2): 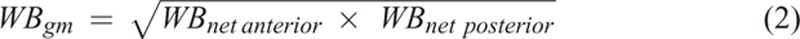


where WB_gm_ is the geometric mean of whole body counts. All geometric mean counts were normalized to the 1st data point to calculate the retention time for the whole body.

### Blood sampling

Blood activity was measured in a calibrated well counter (Capintec CRC-25 Dose Calibrator) with precision of microcurie. Background count was measured without blood samples before measurement of each blood sample activity. Two milliliter blood samples were collected at different time periods postadministration of radioiodine. The 1st measurement was performed approximately 2 hours after radioiodine administration, with further measurements performed after 6, 12, 24, 48, and 96 hours. Similarly, along with whole body measurements, the patients were asked to take the 1st blood sample without maturation, 2 hours after radioiodine administration. Data accuracy was confirmed by quality control methods as recommended in Lassmann et al.^2^ All blood activity was normalized to the administrated activity to calculate the retention per mL of blood.

### Absorbed Dose Calculation

According to the generally accepted MIRD formalism, and also the following formula, recently described by Lassmann et al, the mean absorbed dose to the blood ( 
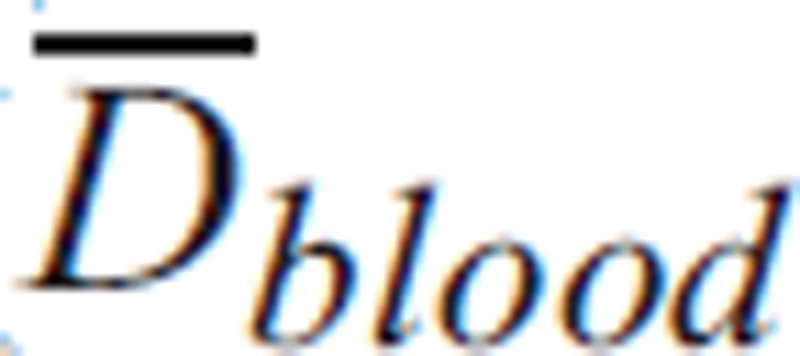
) per unit activity administered (A_0_) was calculated using Eq. (3).^2^ 
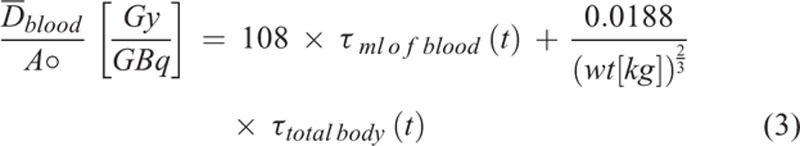


where τ_ml of blood_ and τ_total body_ are the blood and whole body residence times, obtained using the curve fitted to time-activity curve, and wt is the weight of the patient. The function of residence time was described using Eqs. (2) and (4). 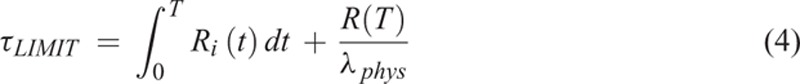


where R_i_(t) is the retention function, i the blood or whole body, and T is the last time the data point is taken and λ_*phys*_ = 0.0036*h*^−1^. The function of activity was calculated using Eqs. (2) and (5): 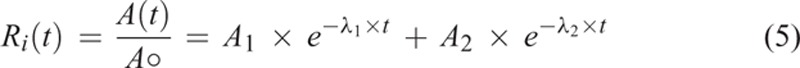


Similarly, where R_i_(t) is the retention function that describing to the function of the administrated activity A_0_ as a function of time t and A_1_, A_2_, λ_1_, and λ_2_ are the fit constants. So, calculating to the fit constants, the bi-exponential curves describing the blood activity and whole body activity as a function of time were plotted separately. Also, the final residence time for whole body and activity in blood, τ_total__body_(t) and τ_ml__of__blood_(t), was calculated by the following Eqs. (2) and (6): 
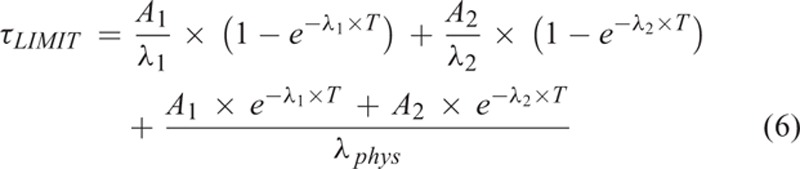


Also, the tolerable activity for a blood absorbed dose of 2 Gy was calculated by Eqs. (2) and (7):(7) 
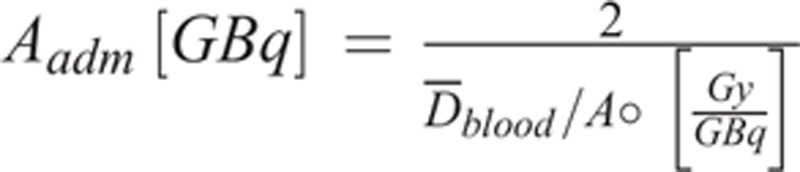


Finally, the correlations between the absorbed dose to blood, blood activity, and whole body data were obtained using SPSS software.

## RESULTS

Twenty three randomly selected patients, suffering from DTC, were enrolled in this study. Mean patients’ age (±SD), height, and weight were 39.3 ± 11.5 years, 161.6 ± 6.3 cm, and 66.3 ± 11.5 kg, respectively. According to their height and weight, blood volume of patients was estimated at 5125.9 ± 302.21 mL for men and 3570.7 ± 473.04 mL for women.^14^

**FIGURE 1:**
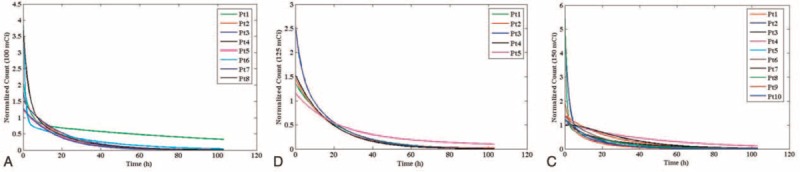
The bi-exponential curves for whole body activities of (A) 3.7 GBq, (B) 4.62 GBq, and (C) 5.55 GBq. As shown all curves show decreasing trend of the whole body count and patients with high level residence time activity to whole body have the larger area under the time-activity curve.

**FIGURE 2:**
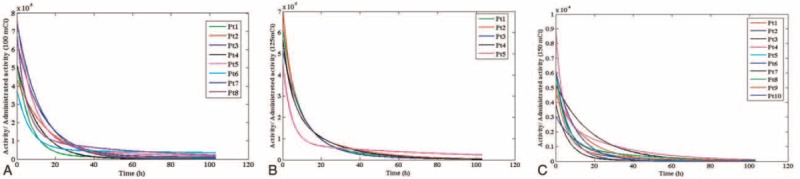
The bi-exponential curves for whole blood activities of (A) 3.7 GBq, (B) 4.62 GBq, and (C) 5.55 GBq. As shown all of curves show decreasing trend of the blood samples activities and patients with high level residence time activity to blood have the larger area under the time-activity curve.

**FIGURE 3:**
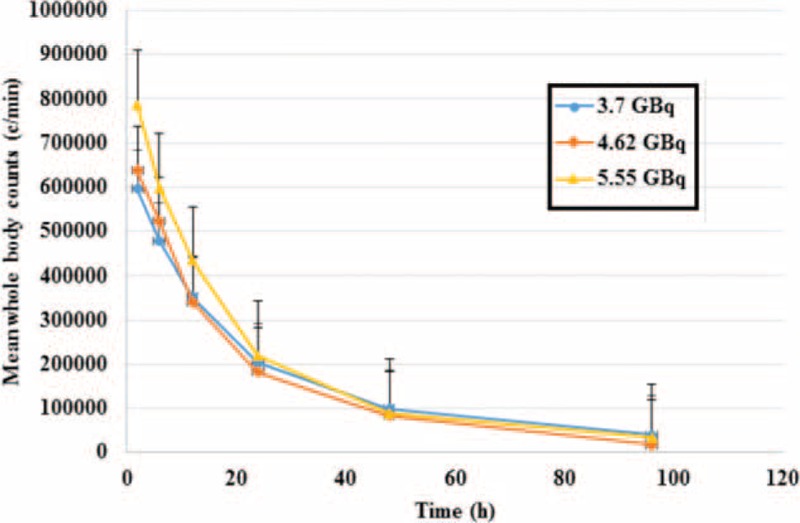
Mean whole body counts versus time for administrated activities at 3.7, 4.62, and 5.55 GBq. The area under the curves for patients with administrated activity of 5.55 GBq are found to be more than other patients, therefore the mean whole body dose to blood are more than the 2 other groups.

**FIGURE 4:**
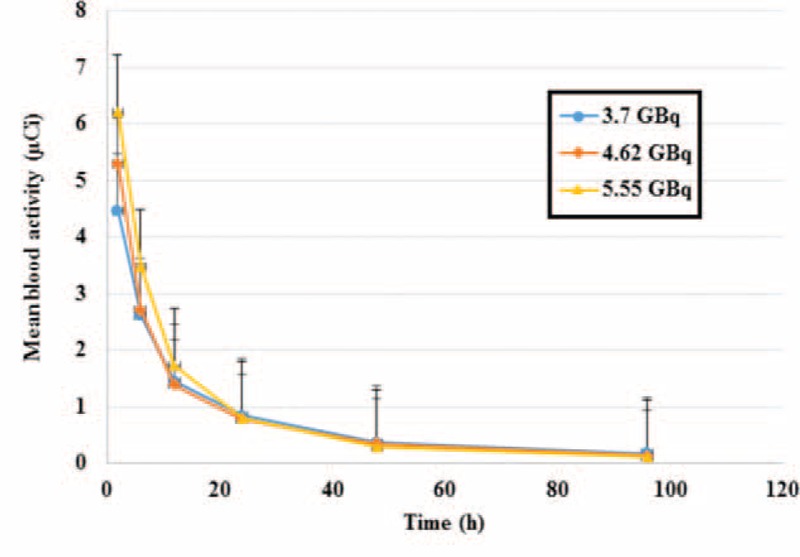
Mean blood activity versus time for administrated activities at 3.7, 4.62, and 5.55 GBq. The area under the curves for patients with administrated activity of 5.55 GBq are found to be more than other patients, therefore the mean blood dose to blood are more than the 2 other groups.

The blood dose measurement was done using whole body scan and blood sampling was performed 6 times after radioiodine administration. The mean whole body counts obtained with administration activities of 3.7, 4.62, and 5.55 GBq at times 2, 6, 12, 24, 48, and 96 hours after radioiodine administration are shown in Table 1. The result of the blood sampling activity obtained for different groups of patients, 2, 6, 12, 24, 48, and 96 hours after radioiodine administration is shown in Table 2. Figure 1 shows the fitted bi-exponential curves for whole body counts of all patients with the different administrated activity. Figure 2 shows the fitted bi-exponential curves for blood counts of all patients with the different administrated activity. The time-activity curves showing mean whole body and the mean blood counts for the different activity at 3.7, 4.62, and 5.55 GBq of radioiodine are shown in Figures 3 and 4, respectively.

**TABLE 1:**
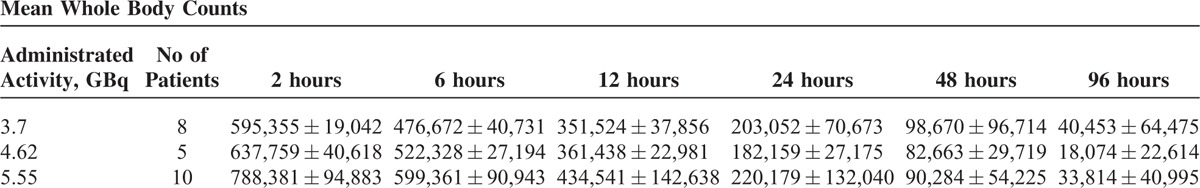
Mean Whole Body Counts After Radioiodine Administration

**TABLE 2:**
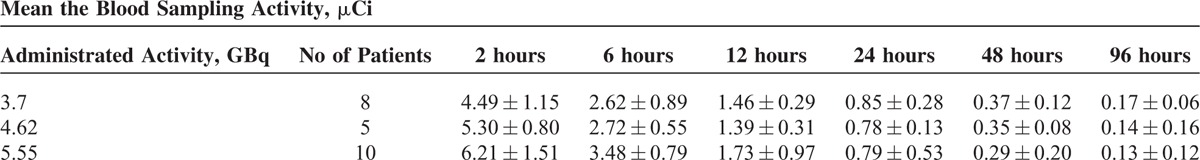
Mean the Blood Sampling Activity After Radioiodine Administration

Figures 3 and 4 show a decreasing trend as it is expected. The mean whole body count and the mean activities of the blood samples for patients with 5.55 GBq activities are found to be more than the 2 other groups. However, they reach the same trend after 24 hours. These parameters for patients with the activity of 4.62 GBq found to be more than those of the patients receiving 3.7 GBq in the 1st 12 hours, and the 2 curves show approximately the same pattern after 12 hours. Comparing the area under the curves shown in Figures 3 and 4, it can be concluded that the mean blood doses for patients with administrated activity of 5.55 GBq are more than the 2 other groups.

According to the results of this study, the absorbed dose to the blood in one of the patients was about 0.3 Gy with administration of 3.7 GBq and about 0.4 Gy with administration of 3.7 and 5.55 GBq in 4 others. In 8 other patients, the blood absorbed dose was 0.5 Gy with administration of 4.62 and 5.55 GBq. The blood absorbed dose for the remaining patients was between 0.5 and 1 Gy with administration of 3.7, 4.62, and 5.55 GBq, and only for one of the patients, the blood dose was about 2 Gy with administration of 5.55 GBq.

Based on Lassmann's equation and the results of whole body counting and blood sample activity, blood dose measurements 96 hours after administration of 3.7, 4.62, and 5.55 GBq of radioiodine absorbed doses to patients’ blood were 0.65 ± 0.18, 0.67 ± 0.20, 0.79 ± 0.51 Gy, respectively.

Data analysis showed that increasing radioiodine activity from 3.7 to 5.55 GBq increases blood dose significantly, while there was no significant difference in blood dose when the radioiodine activities were 3.7 and 4.62 GBq. The mean (±SD) values of absorbed doses to the blood 96 hours after radioiodine administration are shown in Table 3. The results show that no significant difference (*P* value = 0.4) was observed in patient's doses by increasing the radioiodine activity from 3.7 to 4.62 GBq. The results obtained by the statistical software (IBM SPSS Statistics 19) showed significant correlation between the absorbed dose to the blood and whole body data at 24 to 48 hours with correlation coefficient 0.67 and *P* < 0.001. Also the absorbed dose to the blood and blood sampling activity after at least 48 hours postadministration with correlation coefficient 0.62 and *P* < 0.001 had significant correlation.

**TABLE 3:**
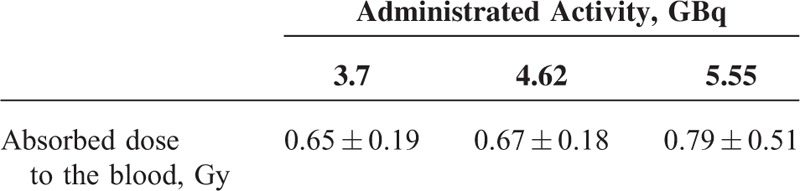
Mean Values of Absorbed Dose to the Blood 96 hours After Radioiodine Administration

## DISCUSSION

The factors affecting the blood absorbed dose are the renal and gastrointestinal functions (urine and stool frequency), fluid intake, presence and extent of metastases and other physiological and pathological iodine uptakes,^15,16^ different lesion uptakes due to the different biological half-life of each patient, and perspiration level. One of the most obvious reasons for increasing blood dose of a patient in comparison with other patients with the same administration activity is high level residence time activity to blood and whole body that produce the area under the time-activity curve and the blood dose. According to the EANM Dosimetry Committee Series protocols on “Standard Operational Procedures for Pre-therapeutic Dosimetry,” the DTC treatment is safe if the absorbed dose to the blood does not exceed 2 Gy.^2^ In recent years, many studies have been performed to find the proper administrated activity of radioiodine for the treatment of DTC.^17–19^ Some studies found that radioiodine therapy is safe if the blood dose is confined to less than 2 Gy, while keeping the whole body retention less than 4.4 GBq at 48 hours, and the pulmonary uptake at 24 hours less than 3 GBq.^7^ Although other studies have different recommendations for radioiodine levels from 1.11 up to 7.4 GBq.^20–22^

Generally, it is expected that the whole body counts and blood activity be maximum at the 1st measurement time. According to Tables 1 and 2, the whole body counts and blood activity without biological disposal are high. As the result demonstrated, other time measurements cannot be estimated because each patient has different biological disposal and renal performance. According to Figure 1, the decreasing trend of the whole body count is a natural process. However in Figure 1A, the decreasing procedure was very slow for patient 1 with administrated activity of 3.7 GBq, which caused the area under the curve, residence time, and absorbed dose to the patient's blood to increase. This situation is observed for patient 5 with administrated activity of 4.62 GBq in the Figure 1B, and for the patients 4 and 7 with administrated activity of 5.55 GBq in the Figure 1C. Similarly, all these are demonstrated at the curves of Figure 2.

This study showed that absorbed dose to the patient's blood with radioiodine activity of administered 4.62 GBq is similar and only for one of the patients with this amount of activity, different absorbed dose was recorded. According to Table 3, no significant difference in absorbed dose to the patient's blood was observed when treated with 3.7 GBq of radioiodine compared to 4.62 GBq. Although there is significant difference in the absorbed dose to the patient's blood with administrated activity of 5.55 GBq compared with that of patients with administrated activity of 3.7 GBq of radioiodine.

In this study, the mean blood absorbed dose was estimated to be 0.65 ± 0.19 Gy with administrated activity 3.7 GBq. The results of the blood dose obtained in this study are compared with the results obtained in previous investigations in Table 4. M’Kacher et al^23^ and Monsieurs et al^24^ estimated the absorbed dose to the blood to be 0.54 and 0.32 Gy, respectively. Also, Watanabe et al^25^ demonstrated that radiation damage to lymphocytes in patients with thyroid cancer after treatment with 3.7 GBq radioiodine is equivalent to the effects observed after a mean dose of 0.45 Gy. Similarly, in another study performed by Hänscheid et al^8^ on a series of patients treated with 3.7 GBq radioiodine, the mean dose to the blood was estimated to be 0.62 Gy. The results showed significant correlation between the absorbed dose to the blood and whole body counts at 24 to 48 hours. Also the absorbed dose to the blood and blood sampling activity after at least 48 hours had significant correlation. The important limitations in this study are the small sample size and limited administrated activities. So, it is necessary that future researches be performed with a larger number of patients and various administrated activities of radioiodine in order to determine more accurate results for absorbed dose to the blood and the optimum activity for the best response to treatment. In the whole body measurements made with the gamma camera, it has been assumed that the administered activities do not result to count rates so high, so as to saturate the detector. For this reason no dead time corrections were applied. This could be another limitation of this study, especially for the patients with the higher administered activities.

**TABLE 4:**
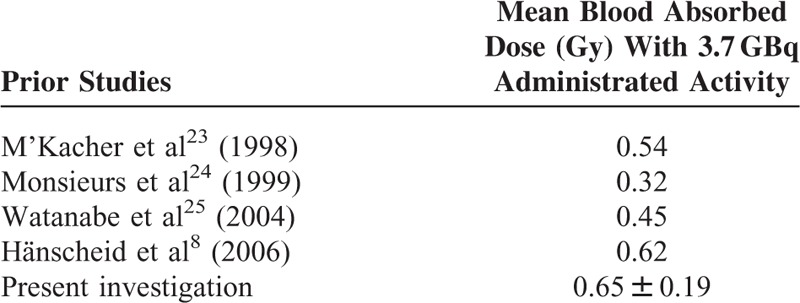
Comparison of Mean Blood Absorbed Dose (Gy) With 3.7 GBq in Previous Investigations

## CONCLUSION

Our results revealed a significant correlation between the absorbed dose to the blood and whole body counts at 24 to 48 hours. Also the absorbed dose to the blood and blood sampling activity after at least 48 hours had significant correlation.
